# Curative-intent pancreas resection for pancreatic metastases: surgical and oncological results

**DOI:** 10.1007/s10585-020-10029-z

**Published:** 2020-02-24

**Authors:** Sophia Chikhladze, Ann-Kathrin Lederer, Christian M. Kühlbrey, Julian Hipp, Olivia Sick, Stefan Fichtner-Feigl, Uwe A. Wittel

**Affiliations:** 1 Department of General and Visceral Surgery, Medical Center - University of Freiburg, Faculty of Medicine, University of Freiburg, Hugstetter Straße 55, 79106 Freiburg im Breisgau, Germany; 2 Center for Complementary Medicine, Department of Environmental Health Sciences and Hospital Infection Control, Medical Center - University of Freiburg, Faculty of Medicine, University of Freiburg, Breisacher Straße 115b, 79106 Freiburg im Breisgau, Germany

**Keywords:** Pancreas surgery, Renal cell carcinoma, Melanoma, Survival, Metastasectomy

## Abstract

**Background:**

Pancreatic metastasis is a rare cause for pancreas surgery and often a sign of advanced disease no chance of curative-intent treatment. However, surgery for metastasis might be a promising approach to improve patients’ survival. The aim of this study was to analyze the surgical and oncological outcome after pancreatic resection of pancreatic metastasis.

**Methods:**

This is a retrospective cohort analysis of a prospectively-managed database of patients undergoing pancreatic resection at the University of Freiburg Pancreatic Center from 2005 to 2017.

**Results:**

In total, 29 of 1297 (2%) patients underwent pancreatic resection due to pancreatic metastasis. 20 (69%) patients showed metastasis of renal cell carcinoma (mRCC), followed by metastasis of melanoma (n = 5, 17%), colon cancer (n = 2, 7%), ovarian cancer (n = 1, 3%) and neuroendocrine tumor of small intestine (n = 1, 3%). Two (7%) patients died perioperatively. Median follow-up was 76.4 (range 21–132) months. 5-year and overall survival rates were 82% (mRCC 89% vs. non-mRCC 67%) and 70% (mRCC 78% vs. non-mRCC 57%), respectively. Patients with mRCC had shorter disease-free survival (14 vs. 22 months) than patients with other primary tumor entities.

**Conclusion:**

Despite malignant disease, overall survival of patients after metastasectomy for pancreatic metastasis is acceptable. Better survival appears to be associated with the primary tumor entity. Further research should focus on molecular markers to elucidate the mechanisms of pancreatic metastasis to choose the suitable therapeutic approach for the individual patient.

## Introduction

P ancreatic metastasis from other primary malignancies is a rare cause for pancreas surgery. Isolated pancreatic metastasis is known to occur in cases of renal and colorectal carcinoma, melanoma, breast and lung cancer [1]. The rate of metastasis to the pancreas is about 2% of all pancreatic malignancies [2–4]. Autopsy records of patients with malignant diseases excluding primary pancreatic cancer revealed that 15% had pancreatic metastasis and, thus, more frequently than clinically diagnosed [5]. It can be assumed that patients with pancreatic metastases are often in an advanced stage of primary disease, and the diagnosis of pancreatic metastasis is an incidental finding as part of follow-up care. Most of these patients present no metastasis-related symptoms [6, 7]. Curative intent of surgery is only given in patients with controlled primary malignant disease, which leads to a small fraction of patients undergoing pancreas resection. The most common primary entity with isolated pancreatic metastasis is renal cell carcinoma (mRCC) showing pancreatic metastasis usually years after diagnosis of the primary tumor [1, 8–10]. Resection of pancreatic metastasis of mRCC was associated with improved patient survival compared to metastasis resection of other primary tumors [1, 8, 11, 12]. Due to the rarity of resectable pancreatic metastases, no guideline has been established for surgical treatment of different tumor entities. Only few data of surgical and oncological treatment have been reported to date from case reports or small sample size studies. The potential advantage and also the risk of pancreatic resection for these patients still remain unclear. The objective of this study was to analyze the surgical and oncological outcome after pancreatic resection of pancreatic metastasis and, therefore, we retrospectively evaluated our own prospectively-collected institutional data of patients who underwent pancreatic resection.

## Methods

Data were retrospectively obtained from the pancreatic surgery database of the University of Freiburg Medical Center, which contains prospectively-collected data for all pancreatic resections performed at our institution. Inclusion criterion was histologically-proven pancreatic metastasis. All kinds of primary tumor entities except primary pancreas carcinoma were eligible for inclusion. Patients with tumor infiltration in the pancreas due to cavitary metastasis were not included. All kinds of surgical approaches were considered. Primary outcome was overall survival of mRCC patients compared to patients with other metastatic tumor entities (nmRCC). Patients’ demographic data including sex, age, comorbidities, time between surgery of primary tumor and pancreatic metastasis, localization of metastasis and extent of tumor, imaging modalities used, kind/extent of surgery, interventions, histology, complications and clinical and oncological outcome were analyzed. Perioperative complications were graded according to the recommendations of the International Study Group of Pancreatic Surgery (ISGPS) criteria [13–15] and the Clavien–Dindo classification [16, 17]. Perioperative mortality was defined as death during the initial hospitalization or within 30 days of the operative date.

Study size depended on feasibility, as pancreatic metastases are rare. Data are presented as median values and their ranges unless otherwise specified. Continuous variables were analyzed using the Mann–Whitney-U test and categorical variables were analyzed using the Chi-squared test or Fisher exact test, as appropriate. Survival analysis was performed using the Kaplan–Meier method. A p value of < 0.05 was considered statistically significant. All statistical analyses were performed with SPSS for Windows (version 25.0, SPSS Inc., Chicago, IL, USA). The study was approved by the Ethics Committee of the University of Freiburg Medical Center.

## Results

We retrospectively analyzed data of 1297 patients undergoing pancreatic resection between 01/2005 and 12/2017. Only 2% of those (n = 29) underwent pancreatectomy for pancreatic metastatic disease. All patients were operated with curative intent. The median age of all patients was 66 years (range 44–79). More than half of the patients (n = 16, 55%) were male. In two-thirds of the patients (n = 19, 66%) pancreatic metastasis was diagnosed as a part of follow-up care. Only 10 of 29 patients (34%) had clinical symptoms, which were mostly unspecific (n = 8, 28%). Two patients (7%) showed tumor-related symptoms: One patient (3%) presented with jaundice and another with delayed gastric emptying. The diagnostics of pancreatic lesions included CT-scan in 18 (62%), MRI in 13 (45%), both CT and MRI in 6 (21%) patients and PET-CT in 14 (48%). Since the primary diagnosis was known to the radiologist, sensitivity of imaging could not be definitively assessed. Sensitivity of CT, MRI and PET-CT was 89%, 92% and 71%, respectively. Seventeen patients (59%) showed single metastasis and 12 patients (41%) had on average 3 (range 2–15) metastases in the pancreas. At the time of surgery, 13 patients (45%) had extra-pancreatic metastasis for which curative-intent resection was also planned. 19 (65%) of the patients had a previous history of metastases, and 18 (62%) patients had had on average one resection of metastases (range 0–8 metastatic resections) before resection for pancreatic metastases. Metastases were metachronous in 97% of the patients (n = 28). The majority of metastases were located in the pancreatic tail (n = 8, 28%), followed by the head (n = 6, 21%) and body (n = 6, 21%). Nine patients (31%) had lesions in several localization in pancreas. The majority of pancreatic metastases originated from renal cell carcinoma (n = 20, 69%), mostly from the left kidney (n = 11, 55%). Other primary tumor entities were melanoma (n = 5, 17%), colon cancer (n = 2, 7%), ovarian cancer (n = 1, 3%) and NET of the small intestine (n = 1, 3%). The median tumor size was 21 mm (range 4–60). Seventeen patients (59%) underwent distal pancreatectomy, pancreatic head resection was performed in 6 (21%) and total pancreatectomy in 6 (21%) patients. Splenectomy was performed in 18 patients (62%) and adrenalectomy in three patients (10%). One patient each (3%) underwent liver or gastric resection and further two (6%) underwent small bowel resection. Three patients (10%) underwent laparoscopic pancreas surgery (1 total pancreatectomy and 2 distal pancreatectomies). R0-resection was possible in 25 patients (86%). Median operative time was 281 min (range 154–556). Perioperative blood transfusion was needed in 4 patients (14%). Overall, 21 patients (72%) suffered from postoperative complications. Complications requiring intervention (Clavien-Dindo III–V) were observed in 9 patients (31%) of whom 4 developed organ dysfunction. Clinically-relevant grade B and C pancreatic fistula occurred in 14 patients (61%)**.** Two patients (7%) died during the postoperative course due to liver failure and multi-organ failure due to gastric perforation. The median follow-up time of all patients was 76.4 (range 21–132) months. Overall survival rate was 70% (n = 19). 5-year survival was 82% (n = 22).

### Renal cell carcinoma vs. other tumor entities

Patients were divided into two groups for further analysis: Metastatic renal cell cancer (mRCC) and metastases due to other tumor entities (mNRCC). Patients with mRCC were significantly older (p = 0.02) and, due to past renal resection, more frequently had renal-dependent diseases such as hypertension (p = 0.02) and renal insufficiency (p = 0.002). ASA score was slightly but non-significantly higher in patients with mRCC. BMI, heart and pulmonary comorbidities and diabetes were similar in both groups (Table 1). Before pancreas surgery, patients with mRCC had no other therapy (radiation, chemotherapy or targeted therapy) than surgery and RFA, whereas patients with nmRCC had several other therapies [surgery in 8 cases (89%), chemotherapy in 3 cases (33%), radiation in 3 cases (33%) and interferon therapy in 5 cases (56%)]. Diagnostic methods differed significantly between the groups (Table 2, p = 0.014). Localization of pancreatic metastases was similar in both groups with the exception of multiple metastases, which were only found in mRCC patients (n = 9, Table 2). The median time between resection of primary tumor and pancreatic metastasis was 116 months (range 6–331 months) for mRCC and 66 months (range 13–108 months) for other tumor entities (p = 0.07). Perioperative blood transfusion was only needed in patients with mRCC. Development of postoperative complications was similar in both groups (mRCC 70% vs. nmRCC 78%, Table 3). Clinically-relevant postoperative pancreatic fistula (POPF grade B and C) occurred in 9 (64%) mRCC patients and in 5 (55%) mnRCC patients (rate excludes patients with total pancreatectomy). Patients with mRCC stayed slightly but non-significantly longer in the intensive care unit and in hospital (Table 3). All postoperatively deceased (n = 2, 7%) were patients with mRCC.

**Table 1:** Demographic data, comorbidities and survival

Parameter	All (n = 29)	mRCC (n = 20)	mnRCC (n = 9)	p
Sex [n (%)]	♀13 (45%)	♀10 (50%)	♀3 (33%)	NS
	♂16 (55%)	♂10 (50%)	♂6 (67%)	
Age [years, median (range)]	66 (44–79)	68 (53–78)	63 (45–74)	**0.02**
ASA-Score [n (%)]				NS
I	0 (0%)	0 (0%)	0 (0%)	
II	17 (59%)	10 (50%)	7 (78%)	
III	11 (38%)	9 (45%)	2 (22%)	
IV	1 (3%)	1 (5%)	0	
BMI [kg/m^2^, median (range)]	28 (20–41)	29 (20–41)	23 (20–34)	NS
Diabetes [n (%)]	3 (10%)	2 (10%)	1 (11%)	NS
Hypertension [n (%)]	14 (48%)	12 (60%)	2 (22%)	**0.02**
CHD [n (%)]	1 (3%)	0 (0%)	1 (11%)	NS
Pulmonary disease [n (%)]	10 (34%)	7 (35%)	3 (33%)	NS
Renal disease [n (%)]	17 (59%)	16 (80%)	1 (11%)	**0.002**
Postoperative mortality [n (%)]	2 (7%)	2 (10%)	0 (0%)	NS
Disease-free survival [months, median (range)]^+^	14 (2–170)	14 (2–150)	22 (4–170)	NS
Time from primary tumor to metastasis [months, median (range)]	89 (6–331)	116 (6–331)	66 (13–108)	**0.07**
Metachronous diagnosis [n (%)]	28 (97%)	20 (100%)	8 (89%)	NS
5-year survival rate [n (%)]*	22 (82%)	16 (89%)	6 (67%)	NS
Overall survival rate [n (%)]*	19 (70%)	14 (78%)	5 (56%)	NS

*CHD* coronary heart disease, *y* years, *mRCC* Metastatic renal cell carcinoma, *mnRCC* Metastatic other tumor entities

^+^Five patients without R0-Resection excluded *Two postoperatively-deceased RCC patients excluded

**Table 2:** Tumor-specific and diagnostic data

Parameter	All (n = 29)	mRCC (n = 20)	mnRCC (n = 9)	p
Localization of tumor [n (%)]				NS
Head	6 (21%)	3 (15%)	3 (33%)	
Body	6 (21%)	3 (15%)	3 (33%)	
Tail	8 (28%)	5 (25%)	3 (33%)	
More than one localization	9 (31%)	9 (45%)	0 (0%)	
Advance of disease [n (%)]				NS
Single metastasis	17 (59%)	8 (40%)	9 (100%)	
Further pancreatic metastasis	12 (41%)	12 (60%)	0 (0%)	
Extra-pancreatic metastasis	13 (45%)	8 (40%)	5 (56%)	
Past extra-pancreatic metastasis	19 (65%)	12 (60%)	7 (78%)	
Tumor size [mm, median (range)]	21 (4–60)	19 (4–50)	32 (18–60)	NS
Initial tumor stage				NS
T1	8 (27%)	7 (35%)	1 (11%)	
T2	4 (14%)	3 (15%)	1 (11%)	
T3	6 (21%)	3 (15%)	3 (33%)	
TX	11 (38%)	7 (35%)	4 (45%)	
Initial lymph node stage				NS
N0	15 (52%)	11 (55%)	4 (44%)	
N1	1 (3%)	0 (0%)	1 (12%)	
NX	13 (45%)	9 (45%)	4 (44%)	
Symptoms [n (%)]				NS
Pain	2 (7%)	1 (5%)	1 (11%)	
Jaundice	1 (3%)	1 (5%)	0 (0%)	
Diagnostic method [n (%)]				**0.014**
CT	18 (62%)	15 (75%)	3 (33%)	
MRI	13 (45%)	10 (50%)	3 (33%)	
CT and MRI	6 (21%)	5 (25%)	1 (11%)	
PET-CT	14 (48%)	8 (40%)	6 (67%)	
Diagnostic sensitivity				n/a
CT	89%	93%	67%	
MRI	92%	90%	100%	
PET-CT	71%	50%	100%	

*mRCC* Metastatic renal cell carcinoma, *mnRCC* Metastatic other tumor entities

**Table 3:** Operative data and postoperative complications

Parameter	All (n = 29)	mRCC (n = 20)	mnRCC (n = 9)	p
Surgical approach [n (%)]				NS
Pancreatic head resection	6 (21%)	3 (15%)	3 (33%)	
Distal pancreatectomy	17 (59%)	11 (55%)	6 (67%)	
Pancreatectomy	6 (21%)	6 (30%)	0 (0%)	
Resection [n (%)]				NS
R0	25 (87%)	17 (85%)	8 (89%)	
R1	3 (10%)	3 (15%)	0 (0%)	
R2	0 (0%)	0 (0%)	0 (0%)	
Rx	1 (3%)	0 (0%)	1 (11%)	
Perioperative blood transfusion [n (%)]	4 (14%)	4 (20%)	0	NS
Operative time [min, median (range)]	281 (154–556)	287 (154–556)	266 (172–527)	NS
ICU stay [days, median (range)]	4 (1–45)	5 (2–35)	3 (1–45)	NS
Hospital stay [days, median (range)]	18 (8–99)	19(11–699)	13 (8–28)	NS
Postoperative complications*[n (%)]				
Overall	21 (72%)	14 (70%)	7 (78%)	NS
2	12 (52%)	8 (40%)	4 (45%)	
3	5 (17%)	2 (10%)	3 (33%)	
4	2 (7%)	2 (10%)	0 (0%)	
5	2 (7%)	2 (10%)	0 (0%)	
POPF [n (%)]^+^				NS
BL	3 (13%)	2 (14%)	1 (11%)	
B	11 (48%)	7 (50%)	4 (44%)	
C	3 (13%)	2 (14%)	1 (11%)	
DGE [n (%)]				
A	5 (17%)	3 (15%)	2 (22%)	NS
B	3 (10%)	3 (15%)	0 (0%)	
PPH [n (%)]	4 (14%)	2 (10%)	2 (22%)	NS
Relaparotomy [n (%)]	5 (17%)	4 (20%)	1 (11%)	NS
Intervention [n (%)]	8 (28%)	6 (30%)	2 (22%)	NS

According to Clavien-Dindo-classification [16, 17]

*BL* biochemical leakage, *DGE* delayed gastric emptying, *ICU* intensive care unit, *mRCC* Metastatic renal cell carcinoma, *mnRCC* Metastatic other tumor entities, *POPF* postoperative pancreatic fistula, *PPH* postpancreatectomy hemorrhage

^+^Rates exclude pancreatectomy

13 patients with extra-pancreatic metastasis underwent further therapy. In 7 patients (54%) extra-pancreatic metastasis were resected concurrently with pancreatic resection. Five patients (38%) received resection at a later time, 2 patients (15%) had radiation and 1 patient (8%) with cerebral metastasis underwent LINAC based radiosurgery (Table 4). 18 patients (12 mRCC and 6 nmRCC) underwent further oncological therapies. Follow-up was not available in two patients. All data are presented in Table 4.

**Table 4:** Oncological data

Pat. #	Sex, age (years)	Primary tumor	Therapy of primary tumor	Previous metastasis	Therapy of previous metastasis	time until pancreas metastasis	Simultaneous extrapancreatic metastasis	Number and localization in pancreas	Pancreas resection	Therapy of simultaneous extrapancreatic metastasis	Recurrence	Further oncological course	Survival (month)
1	m, 67	RCC, L	Nephrectomy	No	–	73	No	2, multiple	DP	–	No	No	26
2	m, 79	RCC, L	Nephrectomy	No	–	331	No	4, tail	DP	–	–	–	–
3	m, 59	RCC, R	Nephrectomy	No	–	179	Adrenal gland L	1, tail	DP	Resection later	Adrenal gland R, supraclavicular	Surgery 2x, RTx3x Sunitinib, Axitinib	47
4	m, 44	NET	No	No	–	–	No	1, head	PD	–	No	No	52
5	m, 66	RCC, L	Nephrectomy	Kidney R	RFA	16	Kidney R	1, tail	DP	RTx	Kidney R	RTx	34
6	f, 51	Melanoma	Excision, cervical LAD Interferon alpha	No	–	13	M. psoas	1, tail	DP	Simultaneous resection	Multiple metastasis	RTx, Ipilimumab	28
7	m, 63	Colon cancer	Resection	Liver	Resection	28	No	1, head	PD	–	Lung	Surgery 3x	70
8	f, 59	RCC, L	Nephrectomy	Kidney re	Organ preserving resektion	252	No	1, tail	DP	–	Kidney re	RFA, organpreserving resection	32
9	f, 57	RCC, R	Nephrectomy re	Lung, adrenal gland	Resection	64	M. psoas	15, multiple	TP	Simultaneous resection	Lung	Sunitinib, Nivolumab, Cabozantinib	33
10	f, 77	RCC, L	Nephrectomy	No		173	No	3, multiple	DP	–	No	No	12
11	m, 48	Melanoma	Excision, interferon alfa	Lung, neck, schoulder	Resection, RTx, interferon alpha	37	No	1, tail	DP			n.a	28
12	f, 70	RCC, L	Nephrectomy	Kidney R, adrenal gland	Resection	6	Adrenal	3, multiple	DP	Simultaneous resection		n.a	102
13	f, 62	RCC, R	Nephrectomy	No	–	137	No	3, multiple	TP		M. obturatorius externus R, M. vastus medialis L	RTx, surgery 2x, Nivolumab	94
14	m, 68	RCC, R	Nephrectomy	Thyroid gland	Resection	199	Kidney	1, head	PD	Organ preserving resection later	No	No	103
15	m, 75	RCC, R	Nephrectomy	No	–	177	No	4, multiple	TP	–	Bone, adrenal, kidney, lung	RTx	24
16	m, 45	Melanoma	Excision, interferon alfa	Axilla	Resection interferon alpha	75	Cerebral, lung	1, corpus	DP	LINAC–Radiosurgery, resection lung	Cerebral lung	RTx CTx	41
17	f, 75	RCC, L	Nephrectomy	No	–	76	No	1, tail	DP	–	Lung, bone	Sunitinib, Nivolumab, RTx	25
18	f, 69	RCC, R	Neprectomy	Thyroid gland parotid gland	Resection	194	Lung	3, corpus	PD	Resection later	Pancreas adrenal gland	Nivolumab	156
19	f, 61	Ovarial cancer	Resection	Diaphragm, lymph nodes	CTx, Resection	81	Spleen, duodenum	1, tail	DP	Simultaneous resection	Lymphnodes	CTx 4x	42
20	m, 72	Colon cancer	Resection	Liver, lung	Resection	108	No	1, corpus	DP	–	No	No	170
21	m, 53	RCC, L	Nephrectomy	Lung, other kidney	Organ preserving resection	156	Kidney, adrenal bone,	2, multiple	DP	Simultaneous resection, RTx later	Lung	No	15
22	f, 74	Melanoma	Excision, Roferon	Axilla elbow,	Excision, RTx, Roferon, CTx	62	Chest wall	1, head	PD	Resection later	Axilla, Epicondilus medialis L, chest wall	Resection 3x, CTx 6x Trametinib, Ixoten, Ipilimumab	16
23	m, 54	RCC, R	Nephrectomy	Lung	Resection	24	No	2, multiple	TP	–	Liver, lung	Sunitinib, CTx 5x	76
24	f, 66	RCC, L	Nephrectomy	Thyroid gland	Resection	96	Renal bed	5, corpus	DP	Simultaneous resection	Pancreas, kidney	Resection	134
25	f, 65	RCC, L	Nephrectomy	Thyroid gland, pancreas	Resection	96	No	1, head	TP		Lumbar spine, kidney re	Resection, RFA, Pembrolizumab, Axitinib	148
26	m, 46	Melanoma	Excision, Interferon alpha	Lymph nodes, bone, cerebral, abdominal wall, jejunum, adrenal	Resection, RTx, Chemotherapy	70	Ileum	1, corpus	DP	Simultaneous resection	Multiple	Ipilimimab, resection 2x, RTx 5x Pembrolizumab, Mekinist	62
27	m, 69	RCC, R	Nephrectomy	Lung, thyroid gland	Resection	256	No	1, head	PD	–	Supraclaviculär	Resection	67
28	f, 78	RCC, R	Nephrectomy	No	–	168	No	1, corpus	DP	–	No	No	102
29	m, 73	RCC, L	Organ preserving resection	Parotid gland	Resection	19	No	3, multiple	TP	–	–	–	–

*f* female, *m* male, *R* Right, *L* Left, *DP* distal pancreatectomy, *LAD* lymphadenectomy, *PD* pancreatoduodenectomy, *TP* total pancreatectomy, *RFA* radiofrequency ablation, *CTx* Chemotherapy, *RTx* Radiation

**Fig. 1:**
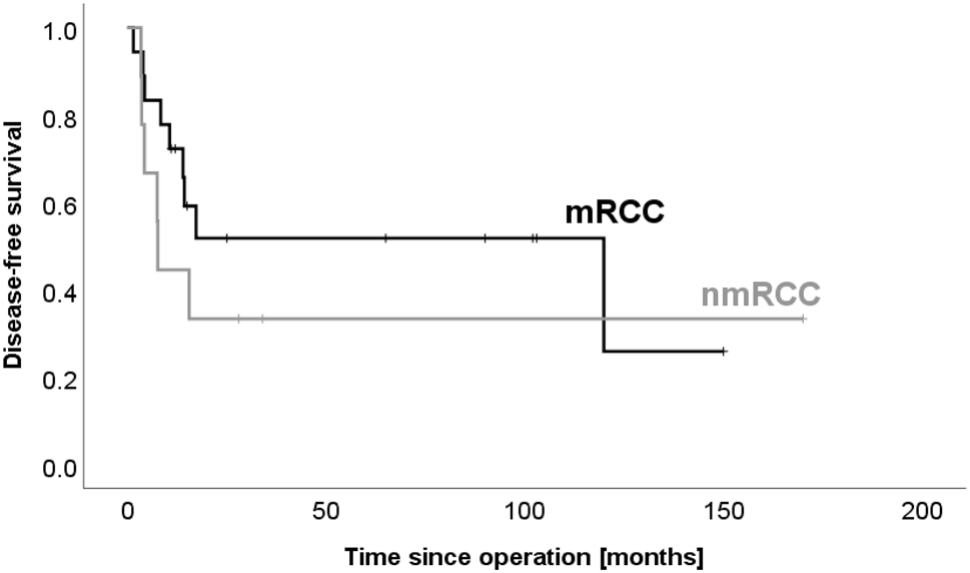
Patients with metastatic renal cell carcinoma (mRCC) might have a shorter disease-free survival than patients with pancreatic metastasis due to other tumor entities (nmRCC) (14 vs. 22 months, p = 0.399)

**Fig. 2:**
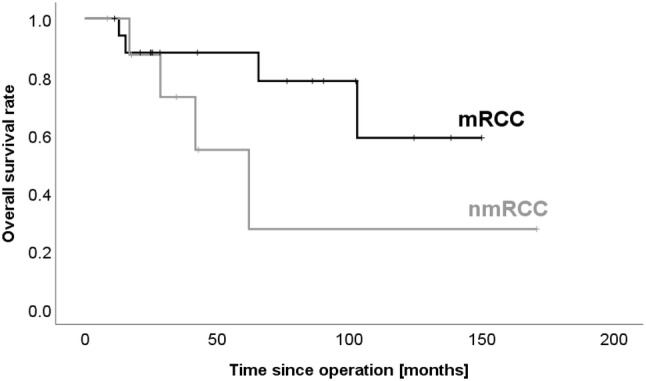
Patients with metastatic renal cell carcinoma (mRCC) might have a better overall survival rate than patients with pancreatic metastasis due to other tumor entities (nmRCC) (78 vs. 56%, p = 0.130)

Interestingly, the disease-free survival appears to be shorter in patients with mRCC than in patients without (14 vs. 22 months, p = 0.399, Fig. 1). 5-year survival was 89% for mRCC (excluding two patients who died postoperatively) and 67% for mnRCC (p = 0.229). Overall survival was 78% and 57% for mRCC and mnRCC, respectively (p = 0.130, Fig. 2). Median survival for mRCC was 54 months (range 11–150), 43 months (range 18–171) for colon and ovarian carcinoma and 28 months (range 8–62) for melanoma.

### Subgroup analysis

Median overall survival was 63.8 (range 15–171) months (n = 18) and 28.4 (range 9–103) months (n = 9) for patients with and without history of previous metastases (p = 0.878), respectively.

Median overall survival was 41.8 (range 8–171) months (n = 14) and 55.7 (range 25–150) months (n = 13) for patients with single and with multiple metastases (p = 0.202), respectively.

Median overall survival was 41.8 (range 8–171) months (n = 17) and 51 (range 11–150) months (n = 10) for patients with single pancreatic and with multiple pancreatic metastases (p = 0.289), respectively.

## Discussion

Patients with secondary metastatic pancreatic malignancy are rare, usually show widespread disease or the primary tumor has aggressive tumor biology with poor prognosis. Due to advanced disease, therapy of pancreatic metastasis appears to be limited, but recent publications show evidence of survival benefit for patients after metastasectomy. Specific symptoms of pancreatic metastasis are mostly lacking, and diagnosis is often made as part of follow-up care. The symptom rate of our patients was only 34% and only two of our patients showed tumor-related symptoms. Reddy et al. reported an unusually high symptom rate of more than 90%, but most were unspecific such as abdominal pain [18]. The rate reported by Reddy et al. might be attributable to data age, since follow-up care was not yet standardized at that time, leading to a diagnosis of metastatic lesions only in case of symptoms. Other studies indicated symptom rates of approximately 20–60% [3, 9, 10, 19, 20]. Most common symptoms are abdominal pain and weight loss, followed by more specific symptoms such as nausea and vomiting, gastrointestinal bleeding and jaundice. Sperti et al. reported a relief of symptoms after metastasectomy until disease recurrence [1]. Surgical treatment for metastatic diseases of liver and lung is well-established, but treatment of metastatic disease of other organs such as the pancreas is still under discussion [21]. The morbidity rate of pancreas surgery is still high, as pancreatic fistula occurs in approximately 20–30% of patients [22, 23]. Severe postoperative complications are diagnosed in 20% of patients [24] and the overall morbidity rate remains up to 60% [25]. The morbidity rate of our cohort was even higher, which might be attributable to a small sample-size, patient’ selection and thorough documentation of complications. The high rate of pancreatic fistula in our cohort might be attributable to the texture of the pancreas and drain management with drains in place over 21 days. It is known that the occurrence of pancreatic fistula depends on the texture of pancreas and that soft texture, which is expected in our cohort to be different than in pancreatic ductal adenocarcinoma, is associated with an increased POPF rate [26]. Furthermore, most of our patients received distal pancreatectomy, which is also known to be associated with a higher POPF rate [27]. These are also confirmed by our results, as we found a lower rate of POPF (B and C) rate in our patents, which underwent pancreatic resection due to pancreatic ductal adenocarcinoma. POPF B and C Rate was here 9% in patients after pancreatoduodenectomy and 20% in patients after distal pancreatectomy. In addition, a smaller pancreatic duct diameter is associated with an increased risk of POPF [28], which is to expect in patients with pancreatic metastasis unlike pancreatic ductal adenocarcinoma.

Once surgical complications have been overcome, survival rate appears to be significantly higher in resected patients [20, 29]. Results of our analysis present the justifiable possibility of curative-intended pancreas surgery in selected patients confirming data previously published by others. Our data are not able to prove superiority of surgery over other therapies as we have no comparable data about a non-surgically-treated group. However, despite advanced tumor disease 70% of our patients were alive at the end of follow-up, which is approximately ten times higher than the 5-year survival rate of patients with pancreatic ductal adenocarcinoma [30]. Selection criteria for our patients who were eligible for surgery of pancreatic metastasis, were fully resectable pancreatic metastasis and an overall low-to-medium surgical risk assessed by a combination of pre-existing illnesses and the current condition. The kind and frequency of pretreatment and the previously-diagnosed tumor entity was not crucial for decision. The most frequently diagnosed tumor origin was mRCC in our cohort. mRCC is known to be the most common primary tumor for pancreatic metastasis [8, 11, 12]. Recent publications have indicated a survival benefit for patients with metastatic pancreatic malignancy from renal cell cancer compared to other primary cancers [1, 11, 31, 32]. Our results confirm this. Even if patients with mRCC had a slightly higher ASA Score and more pre-existing illnesses, the 5-year survival of these patients was higher compared to patients with mnRCC (89 vs. 67%). Interestingly, the disease-free survival was shorter in patients with mRCC compared to mnRCC patients, as was also reported by others [8, 33]. The influence of different tumor pathological types on survival is well-known. The 5-year survival rate for malignancies from melanoma, sarcoma, breast cancer, and colorectal cancer is 20%, 32%, 34%, and 42%, respectively [8, 34]. The survival rate of our mnRCC patients is nearly twice as high as the other reported rates. The observed rate of nearly 90% in mRCC patients is also impressively high, given that the expected 5-year survival for extended disease is less than 20% [35]. These observations are not only attributable to patient selection, but also to the advantage of improved follow-up care leading to a symptom-free, earlier diagnosis, as it is known that symptomatic pancreatic metastasis is associated with decreased survival [33]. In addition, new targeted therapies after surgery are able to significantly improve overall survival of patients [36]. Furthermore, the impressively high survival of advanced-stage cancer patients, the rareness and latency of pancreatic metastasis speak for an underlying exceptional tumor biology. Confirming these hypotheses, most of the patients had a more than 5-year disease-free interval between primary diagnosis and diagnosis of pancreatic lesions, which is also supported by others’ data [37, 38]. Late recurrence of RCC has been known for decades [39], but the pancreas might be a special localization for recurrent cancer disease not only of RCC. In our cohort, the time from primary diagnosis to metastasis was nearly twice as long in mRCC patients compared to nmRCC patients. Furthermore, multiple metastasis of pancreas was only found in mRCC patients, which has also been reported by others [8, 33]. Thus, the origin of metastases could not only determine prognosis but also the intraoperative extent of resection. The prognosis of patients with advanced disease might be also influenced by pancreatic metastasis: Diagnosis of pancreatic metastasis had a positive impact on survival of patients treated with molecular targeted therapies [34, 37, 38, 40]. Molecular targeted therapies such as sunitinib are associated with side-effect rates up to 50%, and early disruption of therapy might worsen the outcome of treated patients with mRCC [41, 42]. Motzer et al. reported a progression-free survival of mRCC patients with sunitinib of 11 months, which is slightly lower than the observed disease-free survival of 14 months in our patients after pancreatic surgery [43]. Another study indicated an even lower survival of less than 6 months [44]. But it must be taken into account that both studies observed all patients with mRCC, not only those with pancreatic metastases. In our cohort, the disease-free survival of 4 patients with pancreatic and extra-pancreatic metastases was only 6 months, but the impact of the result is limited and should lead to further research into whether surgical or non-surgical treatment is the more promising approach for advanced mRCC. Grassi et al. reported that local treatment of pancreatic metastasis, mostly surgery, might be more promising than targeted therapy in mRCC patients [38]. For tumor entities other than RCC, it remains also largely unclear whether surgical or non-surgical therapy is the more promising approach. Ollila et al. observed that median survival in patients undergoing curative resection due to gastrointestinal melanoma metastasis was significantly better than in those undergoing palliative procedures and nonsurgical interventions (49 vs. 5 and 6 months, respectively) [45]. Nevertheless, eligibility for pancreas resection has to be critically evaluated, as pancreas surgery has high morbidity rates and life-threatening complications [25].

Retrospective analyses are limited by lack of documentation and documentation errors. Due to the rareness of pancreatic metastasis, sample-size of our cohort study is really small making it hard to deliver valid results. We were able to obtain data of 29 patients, which is a bigger number than other monocentric analyses have provided. However, the cohort of patients is inhomogeneous due to different tumor entities and different pre-treatments, which makes it hard to compare patient data. Subgroup analysis is also not a promising approach for data comparison, as subgroups are even smaller. Research about rare entities is always limited due to small cohorts, nevertheless, our results are interesting and show tendencies on which to base further research. We were not able to provide data of molecular markers as they were not measured in most of our patients, but we will put more emphasis on these in future research. Due to the scientific advances of the last decade, further research must focus on molecular markers of patients with pancreatic metastasis to identify patients who will benefit from pancreas surgery.

## Conclusion

Pancreas resections appear to be successful in treating patients with advanced cancer with pancreatic metastases. Survival is associated with the histology of primary tumor. Further research is necessary to compare surgical and non-surgical approaches. Focus should be placed on molecular markers to elucidate the mechanisms of pancreatic metastasis in order to choose the therapeutic approach suitable for the individual patient.

## Data Availability

The datasets used and analyzed during the current study are available from the corresponding author on reasonable request.

## Abbreviations

mRCC: Metastatic renal cell carcinoma/cancer
mnRCC: Metastatic other tumor identities
POPF: Postoperative pancreatic fistula

## References

[1] Sperti C, Moletta L, Patanè G (2014). Metastatic tumors to the pancreas: the role of surgery. World J Gastrointest Oncol.

[2] Sperti C, Pasquali C, Liessi G (2003). Pancreatic resection for metastatic tumors to the pancreas. J Surg Oncol.

[3] Niess H, Conrad C, Kleespies A (2013). Surgery for metastasis to the pancreas: is it safe and effective?. J Surg Oncol.

[4] Stankard CE, Karl RC (1992). The treatment of isolated pancreatic metastases from renal cell carcinoma: a surgical review. Am J Gastroenterol.

[5] Nakamura E, Shimizu M, Itoh T, Manabe T (2001). Secondary tumors of the pancreas: clinicopathological study of 103 autopsy cases of Japanese patients. Pathol Int.

[6] Strobel O, Buechler MW (2015). Pancreatic metastases from tumors in the urogenital tract. Gastrointest Tumors.

[7] Strobel O, Hackert T, Hartwig W (2009). Survival data justifies resection for pancreatic metastases. Ann Surg Oncol.

[8] Masetti M, Zanini N, Martuzzi F (2010). Analysis of prognostic factors in metastatic tumors of the pancreas: a single-center experience and review of the literature. Pancreas.

[9] Tosoian JJ, Cameron JL, Allaf ME (2014). Resection of isolated renal cell carcinoma metastases of the pancreas: outcomes from the Johns Hopkins Hospital. J Gastrointest Surg.

[10] Sellner F, Tykalsky N, De Santis M (2006). Solitary and multiple isolated metastases of clear cell renal carcinoma to the pancreas: an indication for pancreatic surgery. Ann Surg Oncol.

[11] Adler H, Redmond CE, Heneghan HM (2014). Pancreatectomy for metastatic disease: a systematic review. Eur J Surg Oncol.

[12] Sperti C, Pozza G, Brazzale AR (2016). Metastatic tumors to the pancreas: a systematic review and meta-analysis. Minerva Chir.

[13] Bassi C, Marchegiani G, Dervenis C (2017). The 2016 update of the International Study Group (ISGPS) definition and grading of postoperative pancreatic fistula: 11 Years After. Surgery.

[14] Wente MN, Veit JA, Bassi C (2007). Postpancreatectomy hemorrhage (PPH): an international study group of pancreatic surgery (ISGPS) definition. Surgery.

[15] Wente MN, Bassi C, Dervenis C (2007). Delayed gastric emptying (DGE) after pancreatic surgery: a suggested definition by the International Study Group of Pancreatic Surgery (ISGPS). Surgery.

[16] Dindo D, Demartines N, Clavien P-A (2004). Classification of surgical complications. Ann Surg.

[17] Clavien PA, Barkun J, de Oliveira ML (2009). The Clavien-Dindo classification of surgical complications: five-year experience. Ann Surg.

[18] Reddy S, Edil BH, Cameron JL (2008). Pancreatic resection of isolated metastases from nonpancreatic primary cancers. Ann Surg Oncol.

[19] Dong J, Cong L, Zhang T-P, Zhao Y-P (2016). Pancreatic metastasis of renal cell carcinoma. Hepatobiliary Pancreat Dis Int.

[20] Zerbi A, Ortolano E, Balzano G (2008). Pancreatic metastasis from renal cell carcinoma: which patients benefit from surgical resection?. Ann Surg Oncol.

[21] Untch BR, Allen PJ (2014). Pancreatic metastasectomy: the memorial sloan-kettering experience and a review of the literature. J Surg Oncol.

[22] Pedrazzoli S (2017). Pancreatoduodenectomy (PD) and postoperative pancreatic fistula (POPF). Medicine (Baltimore).

[23] Bassi C, Dervenis C, Butturini G (2005). Postoperative pancreatic fistula: an international study group (ISGPF) definition. Surgery.

[24] Wellner UF, Keck T (2017). Quality indicators in pancreatic surgery: lessons learned from the German DGAV StuDoQ|pancreas registry. Visc Med.

[25] Ho C, Kleeff J, Friess H, Büchler MW (2005). Complications of pancreatic surgery. HPB.

[26] Peng Y-P, Zhu X-L, Yin L-D (2017). Risk factors of postoperative pancreatic fistula in patients after distal pancreatectomy: a systematic review and meta-analysis. Sci Rep.

[27] McMillan MT, Christein JD, Callery MP (2016). Comparing the burden of pancreatic fistulas after pancreatoduodenectomy and distal pancreatectomy. Surgery.

[28] Hu B-Y, Wan T, Zhang W-Z, Dong J-H (2016). Risk factors for postoperative pancreatic fistula: analysis of 539 successive cases of pancreaticoduodenectomy. World J Gastroenterol.

[29] Mousa OY, Shah R, Hajar N, Landas SK (2015). Periampullary and pancreatic metastases of renal cell carcinoma: an underdiagnosed event. World J Oncol.

[30] Hirshberg Foundation For Pancreatic Cancer Research (2019) Prognosis. https://pancreatic.org/pancreatic-cancer/about-the-pancreas/prognosis/. Accessed 16 Jul 2019

[31] Schwarz L, Sauvanet A, Regenet N (2014). Long-term survival after pancreatic resection for renal cell carcinoma metastasis. Ann Surg Oncol.

[32] Konstantinidis IT, Dursun A, Zheng H (2010). Metastatic tumors in the pancreas in the modern era. J Am Coll Surg.

[33] Chua TC, Petrushnko W, Mittal A (2016). Pancreatic metastasectomy—an analysis of survival outcomes and prognostic factors. J Gastrointest Surg.

[34] Deutsch GB, Flaherty DC, Kirchoff DD (2017). Association of surgical treatment, systemic therapy, and survival in patients with abdominal visceral melanoma metastases, 1965–2014: relevance of surgical cure in the era of modern systemic therapy. JAMA Surg.

[35] Lane BR, Kattan MW (2008). Prognostic models and algorithms in renal cell carcinoma. Urol Clin North Am.

[36] Bai X, Fisher DE, Flaherty KT (2019). Cell-state dynamics and therapeutic resistance in melanoma from the perspective of MITF and IFNγ pathways. Nat Rev Clin Oncol.

[37] Shimizu Y, Iguchi T, Tamada S (2018). Oncological outcomes classified according to metastatic lesions in the era of molecular targeted drugs for metastatic renal cancer. Mol Clin Oncol.

[38] Grassi P, Doucet L, Giglione P (2016). Clinical impact of pancreatic metastases from renal cell carcinoma: a multicenter retrospective analysis. PLoS ONE.

[39] McNichols DW, Segura JW, DeWeerd JH (1981). Renal cell carcinoma: long-term survival and late recurrence. J Urol.

[40] Grassi P, Verzoni E, Mariani L (2013). Prognostic role of pancreatic metastases from renal cell carcinoma: results from an italian center. Clin Genitourin Cancer.

[41] Dreicer R (2009). Management of side effects associated with sunitinib therapy for patients with renal cell carcinoma. Onco Targets Ther.

[42] Ishiyama R, Ishihara H, Kondo T (2019). Negative effect of immediate sunitinib interruption on survival in patients with metastatic renal cell carcinoma. Vivo (Brooklyn).

[43] Motzer RJ, Hutson TE, Tomczak P (2007). Sunitinib versus interferon alfa in metastatic renal-cell carcinoma. N Engl J Med.

[44] Ghosn M, Eid R, Hamada E (2019). OSSMAR: an observational study to describe the use of sunitinib in real-life practice for the treatment of metastatic renal cell carcinoma. J Glob Oncol.

[45] Ollila DW, Essner R, Wanek LA, Morton DL (1996). Surgical resection for melanoma metastatic to the gastrointestinal tract. Arch Surg.

